# Inhibiting CD44-ICD Attenuates LPS-Induced Initiation of Hepatic Inflammation in Septic Mice

**DOI:** 10.3390/ijms25168907

**Published:** 2024-08-15

**Authors:** Li-Hsuan Li, Dur-Zong Hsu, Victor Raj Mohan Chandrasekaran, Ming-Yie Liu

**Affiliations:** Department of Environmental and Occupational Health, College of Medicine, National Cheng Kung University, Tainan 70428, Taiwan

**Keywords:** CD44-ICD, LPS, hepatic inflammation, sepsis, NFkB signaling pathway

## Abstract

Sepsis is a severe condition induced by microbial infection. It elicits a systemic inflammatory response, leading to multi-organ failure, and the liver, as a scavenger, plays a significant role in this process. Controlling hepatic inflammation and maintaining liver function is crucial in managing sepsis. CD44-ICD, as a CD44 signal transductor, is involved in multiple inflammatory responses. However, the role of CD44-ICD in lipopolysaccharide (LPS)-induced hepatic inflammation has not been investigated. Therefore, we aimed to examine whether CD44-ICD initiates hepatic inflammation in septic mice. We induced hepatic inflammation in mice by administering LPS. DAPT, a CD44-ICD inhibitor, was given to mice or Chang cells 30 min or 1 h before LPS administration (10 mg/kg, i.p., or 100 ng/mL, respectively). Inhibition of CD44-ICD decreased the level of aspartate aminotransferase (AST), alanine aminotransferase (ALT), hepatic necrosis, inflammatory cell infiltration, interleukin (IL)-1β, inducible NO synthase (iNOS), nitric oxide (NO) production, nuclear factor (NF)κB signaling pathway proteins, and CD44 expression in mice. CD44-ICD inhibition also decreased IL-1β and CD44 expression levels in Chang cells. CD44-ICD may be a primary regulatory function in CD44-associated LPS-induced initiation of hepatic inflammation in mice.

## 1. Introduction

Sepsis is a systemic dysfunctional host reaction to microbial infection [1,2]. Approximately 31.5 million people are diagnosed with sepsis each year, making it one of the major causes of death in intensive care units [3]. In the pathogenesis of sepsis, inflammation plays a crucial role, as the NF-κB signaling pathway is activated to scavenge the pathogen, producing pro-inflammatory cytokines, tumor necrosis factor (TNF)-α, interleukin (IL)-1β, IL-6, and nitric oxide (NO) [4,5]. Nonetheless, the overproduction of cytokines can lead to tissue injury and organ failure, resulting in the death of patients [6,7,8].

The liver is one of the essential organs in the sepsis-induced inflammatory response because of its role in fighting against bacteria invasion and clearing pathogens [9,10]. During sepsis, the liver acts as a filter, removing toxins and bacteria from the blood, and this process is conducted by the reticuloendothelial system [11]. Kupffer cells, macrophages in the liver, clear pathogens from the bloodstream by endocytosis and phagocytosis and also release pro-inflammatory cytokines to recruit neutrophils to trap the pathogens [12]. However, liver function is impaired due to excessive sepsis-induced inflammation; bacteria can easily spread to other organs via the bloodstream, leading to septic shock and multi-organ failure [11]. In addition, liver dysfunction is highly correlated to septic shock mortality [13]. Therefore, maintaining liver function is crucial in managing sepsis and preventing severe complications such as septic shock and multi-organ failure. Previous studies show that timing is a crucial factor correlated to the mortality of septic patients; the administration of broad-spectrum antibiotics and fluid is positively related to mortality reduction [14,15].

CD44 is a transmembrane adhesion molecule involved in multiple physiological responses, including inflammation, leukocyte rolling, and regulating hyaluronan (HA) and cancers [16,17,18,19,20,21,22]. CD44 has been reported to be involved in different types of inflammation including lipopolysaccharide (LPS)-induced inflammatory response [23,24], non-infection tissue damage-induced inflammation [25], and high-fat diet-induced hepatic steatosis [26]. It comprises three parts: the extracellular domain, transmembrane domain, and intracellular domain (ICD). CD44-ICD acts as a CD44 signal transductor that regulates the CD44’s cancer-related response [27,28]. In addition to cancer-related responses, CD44-ICD is also engaged in neural inflammation in injured nerves [29] and regulates NF-κB expression [30]. Interestingly, CD44 has been widely reported to be involved in metabolic homeostasis, cell proliferation and differentiation, and inflammatory response in the liver [31].

Lipopolysaccharide (LPS) is extensively used as a model for sepsis due to its capacity to induce a systemic inflammatory response, a hallmark of sepsis, even in the absence of a live infective organism. Sepsis is characterized by a dysregulated immune response that leads to systemic inflammation and organ dysfunction. The systemic inflammatory response syndrome (SIRS) is central to sepsis, driven by the excessive release of pro-inflammatory cytokines, causing multi-organ damage. LPS, a component of Gram-negative bacteria, effectively mimics this response by binding to Toll-like receptor 4 (TLR4) on immune cells, triggering the NF-κB pathway, and promoting the release of cytokines such as TNF-α, IL-6, and IL-1β [32,33,34]. Recent studies have validated LPS as a model for sepsis, particularly in hepatic inflammation. The liver, a critical organ in the response to sepsis, is both a target and regulator of systemic inflammation. Hepatic dysfunction is a common sepsis complication due to the overwhelming inflammatory response triggered by endotoxins like LPS [35]. LPS administration leads to a cytokine storm and liver injury, closely mirroring the pathology of sepsis in humans [36,37,38,39]. While LPS does not involve a live pathogen, it remains a widely accepted and relevant model for studying the mechanisms of sepsis-induced inflammation and organ injury. Its ability to replicate the systemic inflammatory response and liver dysfunction seen in sepsis underscores its significance in sepsis research [40]. However, whether CD44-ICD is the major regulator that is involved in the LPS-induced inflammatory response in the liver rather than CD44 has never been investigated. Therefore, we used DAPT, a specific CD44-ICD inhibitor, to inhibit CD44-ICD expression and examine the role of CD44-ICD in initiating hepatic inflammation in LPS-induced endotoxemic mice and Chang cells.

## 2. Results

### 2.1. Inhibiting CD44-ICD Attenuated LPS-Induced Hepatic Injury

**Figure 1:**
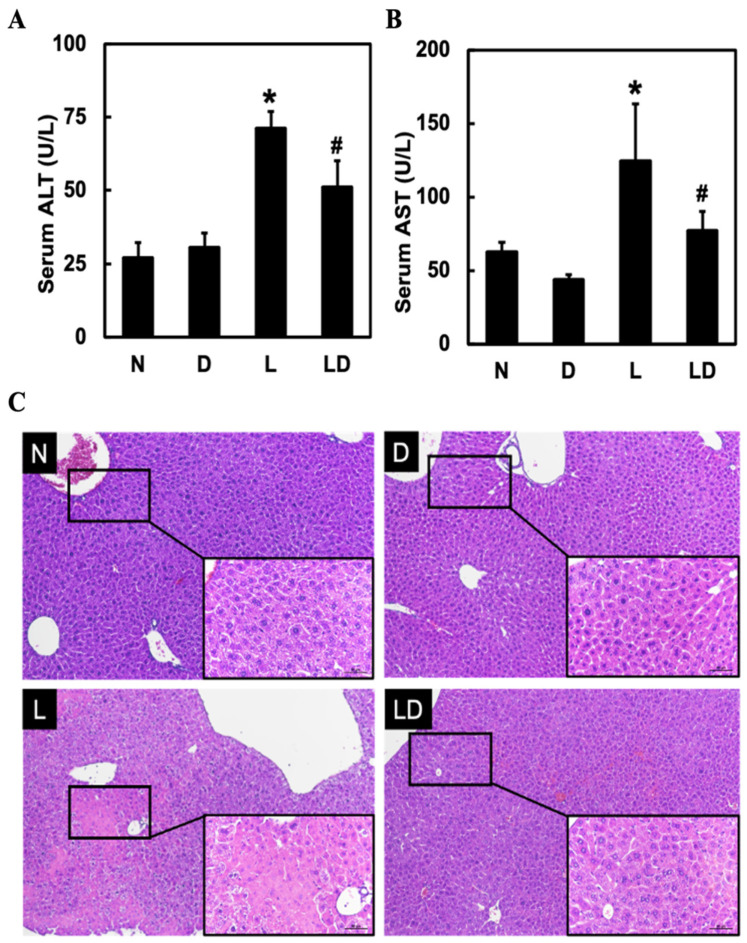
Effects of inhibiting CD44-ICD on LPS-induced hepatic inflammation and histological change in mice. Twenty mice were divided into four groups. N group mice were given only saline; D group mice were given DAPT alone; L group mice were given LPS alone; LD group mice were given DAPT 30 min before LPS. After 6 h, (**A**) AST levels, (**B**) ALT levels in serum, and (**C**) liver histopathological changes (10×, scale bar—100 μm; magnified 20×, scale bar—50 μm) by H&E staining were determined. Data are expressed as mean ± SD (*n* = 5). * *p* < 0.05 compared with N and D groups. # *p* < 0.05 compared with the L group.

To assess the effect of inhibiting CD44-ICD on LPS-induced hepatic injury, we measured the level of AST and ALT and performed H&E staining for histopathological analysis. Twenty mice were divided into four groups: Normal group (N), DAPT alone group (D), LPS alone group (L), and LPS and DAPT group (LD). Serum AST (Figure 1A) and ALT (Figure 1B) were significantly higher in the L group (*p* < 0.05) than in the N or D group and significantly lower in the LD group (*p* < 0.05) than in the L group (Figure 1A,B). Inflammatory cell infiltration and necrosis were found in the L group. However, inflammatory cell infiltration and necrosis were reduced in the LD group (Figure 1C).

### 2.2. Inhibiting CD44-ICD Decreased the Release of Inflammatory Mediators in LPS-Induced Hepatic Inflammation in Mice and Chang Cells

**Figure 2:**
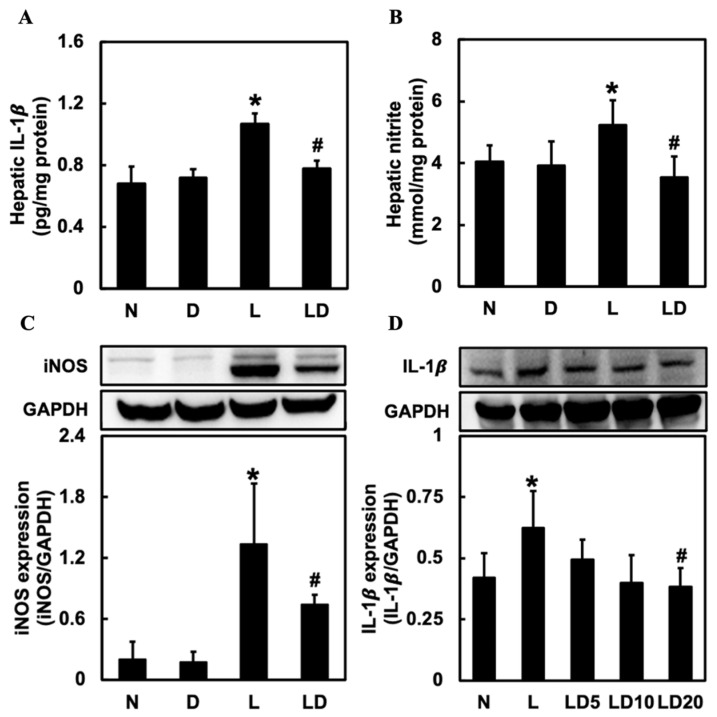
Effects of inhibiting CD44-ICD on pro-inflammatory mediators in LPS-induced hepatic inflammation in mice and cells. Twenty mice were divided into 4 groups. N group mice were given only saline; D group mice were given DAPT alone; L group mice were given LPS alone; LD group mice were given DAPT 30 min before LPS. (**A**) hepatic IL-1β, (**B**) NO, and (**C**) iNOS expressions were determined six hours after LPS. The Chang cells were divided into five groups. N group, cells were treated only DMSO; L group, cells received LPS (100 ng/mL); LD5 group, cells received 5 mM DAPT 1 h before LPS; LD10 group, received 10 mM DAPT 1 h before LPS; LD 20 group, cells were received 20 mM DAPT 1 h before LPS. (**D**) IL-1β levels in Chang cells were determined 6 h after LPS was given. Data are expressed as mean ± SD (*n* = 5). * *p* < 0.05 compared with N and D groups. # *p* < 0.05 compared with the L group.

To determine the effect of inhibiting CD44-ICD on the release of inflammatory mediators in LPS-induced hepatic inflammation in mice and Chang cells, we assessed the level of hepatic IL-1β, NO, and INOS in mice and the level of IL-1β in Chang cells. We used IL-1β to represent inflammatory response in Chang cells because IL-1β could regulate iNOS and NO expression. Hepatic IL-1β, NO, and iNOS were significantly higher in the L group (*p* < 0.05) than in the N or D group and significantly lower in the LD group (*p* < 0.05) than in the L group (Figure 2A–C). Moreover, the level of IL-1β in the L group was significantly (*p* < 0.05) higher than in the N group and significantly (*p* < 0.05) lower in the LD20 group (Figure 2D).

### 2.3. Inhibiting CD44-ICD Downregulated the Expression of NF-κB Signaling Pathway in LPS-Induced Hepatic Inflammation in Mice

**Figure 3:**
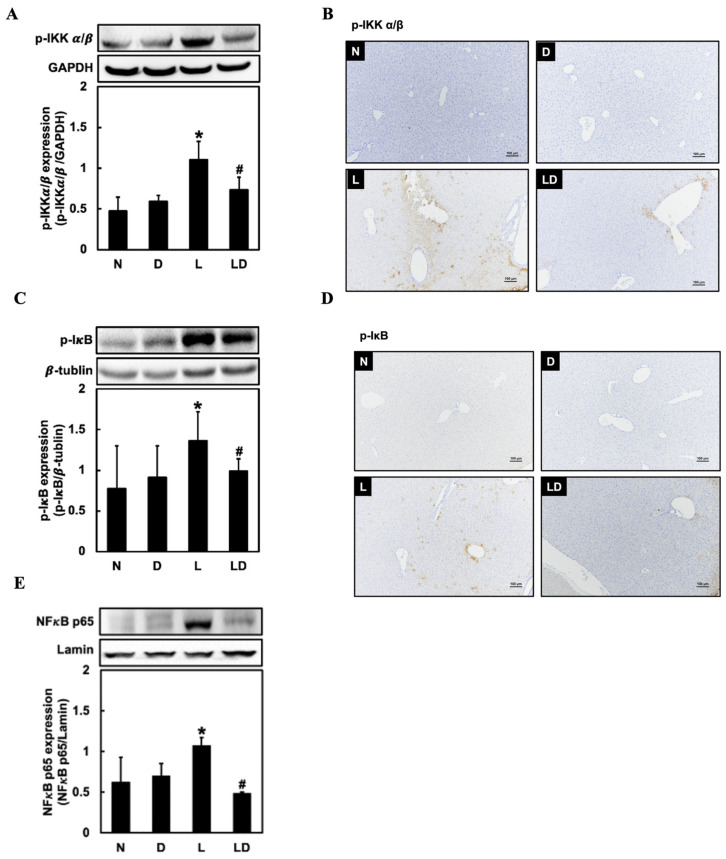
Effects of inhibiting CD44-ICD on NF-κB signaling-related protein expressions in LPS-induced hepatic inflammation in mice. Twenty mice were divided into 4 groups. N group mice were given only saline; D group mice were given DAPT alone; L group mice were given LPS alone; LD group mice were given DAPT 30 min before LPS. (**A**) Western blotting analysis of p-IKK, (**B**) immunohistochemical staining analysis of p-IKK, (**C**) Western blotting analysis of p-IκB, (**D**) immunohistochemical staining analysis of p-IκB, and (**E**) Western blotting analysis of nuclear NF-κB expression in liver tissue was determined 6 h after LPS. The immunohistochemical positive expression were observed under a microscope (10× with a scale bar of 100 μm). Data are expressed as mean ± SD (*n* = 5). * *p* < 0.05 compared with N and D groups. # *p* < 0.05 compared with the L group.

To examine the effect of inhibiting CD44-ICD on the NF-κB signaling protein expressions, we assessed hepatic p-IKK α/β, p-IκB, and nuclear NF-κB expressions. Hepatic p-IKK α/β, p-IκB, and nuclear NF-κB expression was significantly higher in the L group (*p* < 0.05) than in N or D group and significantly lower in the LD group (*p* < 0.05) than in the L group (Figure 3A,C,E). We also assessed the p-IKK α/β, and p-IκB expression by IHC staining, and the result showed that both p-IKK α/β and p-IκB expression in liver tissue was higher than in the N or D group, and lower in the LD group (Figure 3B,D).

### 2.4. Inhibiting CD44-ICD Decreased the CD44 Relative Expressions in LPS-Induced Hepatic Inflammation in Mice and Chang Cells

**Figure 4:**
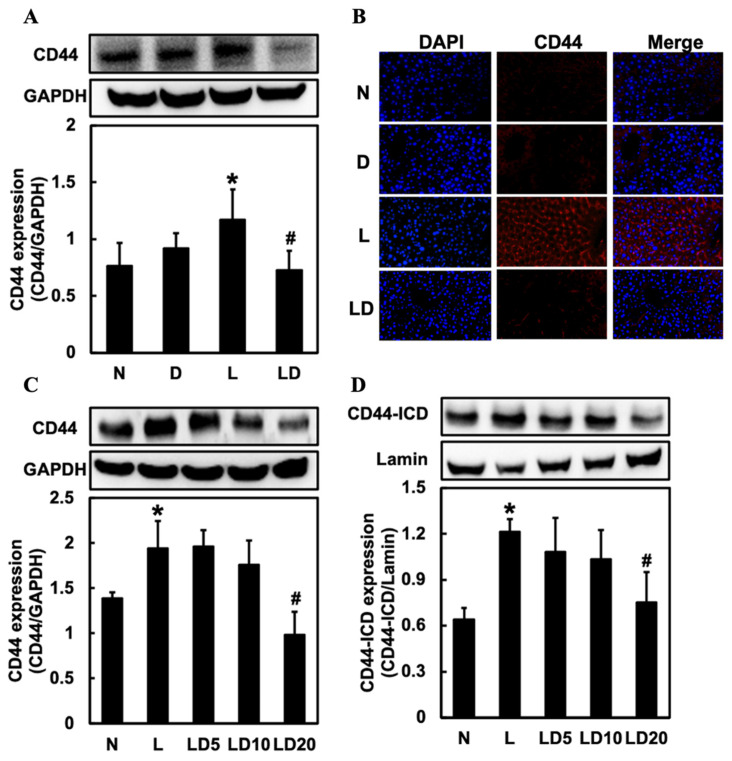
Effects of inhibiting CD44-ICD on CD44 expression in LPS-induced hepatic inflammation in mice and cells. Twenty mice were divided into 4 groups. N group mice were given only saline; D group mice were given DAPT alone; L group mice were given LPS alone; LD group mice were given DAPT 30 min before LPS. (**A**) Western blotting analysis of hepatic CD44 expression and (**B**) immunofluorescence staining analysis of hepatic CD44 expression was determined 6 h after LPS. Positive immunofluorescence reaction for CD44 (red color) and DAPI nucleic acid staining (blue color) was observed (20×) under a microscope. The Chang cells were divided into five groups. N group, cells were treated with only DMSO; L group, cells received LPS (100 ng/mL); LD5 group, cells received 5 mM DAPT 1 h before LPS; LD10 group received 10 mM DAPT 1 h before LPS; LD 20 group, cells received 20 mM DAPT 1 h before LPS. (**C**) CD44 and (**D**) nuclear CD44-ICD expression in Chang cells were determined 6 h after LPS was given. Data are expressed as mean ± SD (*n* = 5). * *p* < 0.05 compared with N and D groups. # *p* < 0.05 compared with the L group.

To investigate the effect of inhibiting CD44-ICD on the CD44 expression, we assessed hepatic CD44 and CD44 and nuclear CD44-ICD expression in Chang cells. Hepatic CD44 and CD44 in Chang cells expressions were significantly (*p* < 0.05) higher in the L group than in the N or D group and significantly (*p* < 0.05) lower in the LD group than in the L group (Figure 4A,C). In the IF staining, we could also see a higher expression of CD44 in the L group and a lower expression in the LD group (Figure 4B). The expression of CD44-ICD was significantly higher in the L group (*p* < 0.05) than in the N group and significantly lower in the LD 20 group (*p* < 0.05) than in the L group (Figure 4D).

## 3. Discussion

We found that inhibiting CD44-ICD might attenuate LPS-induced hepatic inflammation. Inhibiting CD44-ICD reduced LPS-induced hepatic inflammation by decreasing AST and ALT, hepatic injury, IL-1β, NO, iNOS, NF-κB signaling proteins, and CD44 expression.

CD44-ICD may be responsible for LPS-induced hepatic inflammation in experimental septic mice. The liver takes responsibility for scavenging the pathogen from systemic circulation [41,42], and approximately 80% of bacteria will be trapped in the liver after six hours of LPS administration [43]. To defend against the pathogen in the liver, CD44-ICD, as the CD44 signal transductor, will recruit the lymphocyte-recruiting neutrophils to form the structure called neutrophil extracellular traps (NETs) to trap pathogens [9] and upregulate the macrophages’ translocation of NF-κB [44]. Nevertheless, recruiting lymphocytes will interfere with blood flow and cause liver inflammation [11]. If the liver cannot clear all the pathogens and debris, it will cause a more severe inflammatory response, leading to serious liver damage [10]. We found out that the neutrophil infiltration surrounding the central vein and centrilobular necrosis was attenuated by inhibiting CD44-ICD expression. Moreover, because of the reduction in damage to the liver, the levels of AST and ALT were also decreased in LPS-induced hepatic inflammation. Therefore, we hypothesize that CD44-ICD might involve LPS-induced hepatic inflammation in septic mice.

CD44-ICD is involved in endotoxin-initiated hepatic inflammation via NF-κB signaling pathway activation. NF-κB activation was an important transcription factor in the inflammatory response [45]. When inflammation is triggered, IKK and IκB are phosphorylated and activate NF-κB to translocate into the nucleus to release pro-inflammatory cytokines such as TNF-α, IL-1β, IL-6, and iNOS and NO [46,47,48,49]. CD44-ICD promotes the activation of NF-κB to regulate relative inflammatory responses [44]. In the present study, inhibiting CD44-ICD decreased NF-κB translocation, and p-IKK and p-IκB expression. Furthermore, because NF-κB was inhibited, IL-1β, iNOS, and NO were downregulated. As a result, CD44-ICD might, through NF-κB translocation, regulate LPS-induced initiation of hepatic inflammation in septic mice. CD44-ICD might be the significant signal transductor of the CD44-relevant pathway in LPS-induced initiation hepatic inflammation in septic mice. CD44-ICD involves multiple inflammatory responses, neuroinflammation in the injured sciatic nerve, and bladder inflammation [21,29,30]. In addition, CD44-ICD is not just for the transcription factor of CD44 but also regulates the CD44 mRNA level to affect CD44 expression [50]. CD44 is reportedly involved in the LPS-induced inflammatory response by forming a tetramer with TLR4, MD2, and CD14 to initiate the NF-κB signaling pathway [51]. Our study showed that by inhibiting CD44-ICD, the level of CD44 was significantly decreased in LPS-induced hepatic inflammation. Therefore, it may be possible that CD44-ICD plays a major regulatory role in CD44-associated LPS-induced initiation of hepatic inflammation in septic mice. However, further studies are needed to confirm this, and this study has a few limitations: this study primarily utilized the LPS-induced sepsis model, leaving unexplored the role of Toll-like receptor 4 (TLR4) and other regulatory mechanisms, as well as the cecal ligation and puncture (CLP) model, which more closely mimics polymicrobial sepsis. To address these limitations and extend our understanding of the CD44 intracellular domain (CD44-ICD) in sepsis, future research should include in vivo studies using CD44-ICD knockout mice in the CLP model to determine whether CD44-ICD is a critical mediator of the inflammatory response. Additionally, comparative studies between the LPS and CLP models in these knockout mice will help confirm the consistency of CD44-ICD’s role across different sepsis models. At the molecular level, using CD44-ICD knockout cells to measure interleukin-1 beta (IL-1β) levels will further elucidate CD44-ICD’s function in regulating cytokine production. Moreover, investigating TLR4 and other regulatory pathways will provide insights into how CD44-ICD interacts with broader molecular networks during sepsis. Together, these studies will offer a more comprehensive understanding of CD44-ICD’s role in sepsis, highlighting its potential as a therapeutic target.

## 4. Materials and Methods

### 4.1. Chemicals

Lipopolysaccharide (LPS) (derived from *E. coli*, serotype 055:B5) and N-[N-(3,5-Difluorophenacetyl)-L-alanyl]-S-phenylglycine t-butyl ester (DAPT) were purchased from Sigma (St. Louis, MO, USA). Fetal Bovine Serum (FBS) was purchased from Merck (Burlington, MA, USA). Dulbecco’s modified Eagle’s medium (DMEM), penicillin, and streptomycin were purchased from GeneDireX (Taipei, Taiwan, China).

### 4.2. Animal

Male 8–9-week-old C57BL/6 mice were purchased from BioLASCO (Taipei, Taiwan, China). Animals were given a pellet feed diet and water ad libitum. Animals were maintained on a 12h light–dark cycle at a controlled temperature (25 °C). The animal care and experimental protocols followed nationally approved guidelines (IACUC No. 107117).

### 4.3. Chang Cell Line

The human hepatic epithelial cell line Chang was purchased from the American Type Culture Collection (ATCC; Rockville, MD, USA), and cells were cultured in DMEM and supplemented with 10% FBS, penicillin (100 U/mL), and streptomycin (100 U/mL) in a humidified atmosphere of 5% CO_2_ and 95% air at 37 °C.

### 4.4. In Vivo Study

Twenty mice were divided into four groups of five. Normal group (N) received saline; DAPT group (D) received DAPT (10 mg/kg, i.p.); LPS group (L) received LPS (10 mg/kg, i.p.); LPS + DAPT group (LD) received DAPT (10 mg/kg, i.p.) 30 min before LPS (10 mg/kg, i.p). After 6 h, mice were sacrificed by using 2% isoflurane. Hepatic injury was measured by analyzing blood biochemical parameters and histological changes.

### 4.5. In Vitro Study

The Chang cells were divided into five groups (*n* = 5). Each dish was seeded with 1.5 ×10^6^ cells in a 10 mL medium. Normal group (N) received Dimethyl sulfoxide (DMSO); LPS group (L) received LPS (100 ng/mL); LPS + 5 μM DAPT group (LD5), LPS + 10 μM DAPT group (LD10), and LPS + 20 μM DAPT group (LD20) received DAPT 1 h before LPS (100 ng/mL). After 6 h, cells were collected to detect inflammatory relative protein levels. The LPS dose and time point were determined by our preliminary experiments (Supplementary Figure S1A,B).

### 4.6. Blood Collection

Mice blood samples were collected from the inferior vena cava under 2% isoflurane anesthesia. Blood was collected via venipuncture into serum separation tubes, kept for 30 min at room temperature, and then centrifuged at 1000× *g* for 20 min at 4 °C.

### 4.7. Assessment of Hepatic Injury

Hepatic injury was assessed by measuring serum aspartate aminotransferase (AST) and alanine aminotransferase (ALT) levels. Serum samples of 10 μL were dropped on slides and analyzed by using the blood biochemical analyzer (DRI-CHEM 3500s; FUJIFILM, Kanagawa, Japan). Hepatic injury was further assessed by histopathological analysis. Liver tissues were fixed in 10% paraformaldehyde at room temperature for 24 h. Tissues were dehydrated in graded ethanol concentrations, embedded in paraffin, and then cut into 4 μm sections. The sections were stained with hematoxylin and eosin (H&E) for analyzing liver injury.

### 4.8. Measuring IL-1β in Liver Tissue

According to the manufacturer’s instructions, the amounts of IL-1β in liver tissue were determined using an enzyme-linked immunosorbent assay (ELISA) kit (DouSet; R&D Systems, Minneapolis, MN, USA).

### 4.9. Measuring Nitrite Level in Liver Tissue

The amounts of nitrite in liver tissue were measured following the Griess reaction. Liver tissue was homogenized in deionized water (1:10, wt/vol) and centrifuged at 15,000× *g* for 30 min at 4 °C. The supernatant (100 μL) was incubated with 100 μL of Griess reagent at room temperature for 20 min. The absorbance was measured at 550 nm by using a spectrophotometer. Nitrite concentration was calculated by comparing it with a known sodium nitrite concentration standard solution.

### 4.10. Isolating Nuclear Proteins in Liver Tissues and Cells

The tissue samples and cells were homogenized in phosphate-buffered saline (0.1 M, pH 7.4) and were centrifuged at 600× *g* for 5 min at 4 °C. The supernatant was removed, and cytosol extraction buffer A (CEB-A) was added to the tissue pellets (100 μL) and cell pellets (20 μL). After pipetting, the Eppendorf tubes containing tissue and cell homogenate were vortexed for 1 min on the highest setting, then kept on ice for 10 min. The cytosol extraction buffer B (CEB-B) was added to tissue homogenate (5.5 μL) and cell homogenate (1.1 μL), vortexed for 5 s on the highest setting, kept on ice for 1 min, vortexed for 5 s again, and then centrifuged at 16,000× *g* for 5 min at 4 °C. The supernatant was discarded, and the pellet was resuspended with 15 μL of nuclear extraction buffer. The homogenate was vortexed for 1 min, kept on ice for 10 min, and the following steps were repeated four times. The homogenate was centrifuged at 16,000× *g* for 10 min at 4 °C and then transferred the supernatant to the Eppendorf tube. The supernatant contained nuclear proteins stored at −80 °C until use. The amounts of nuclear protein were determined by a nuclear/cytosol fractionation kit (BioVision, Milpitas, CA, USA) according to the manufacturer’s instructions.

### 4.11. Western Blot

The expression levels of IL-1β, iNOS, p-IKK α/β, p-IκB, nuclear NF-κB, and CD44 in liver tissue, and IL-1β, CD44, and nuclear CD44-ICD in cells, were determined using Western blotting. Fifty micrograms of proteins were loaded on 8–15% sodium dodecyl sulfate-polyacrylamide gel (SDS-PAGE), depending on the molecular weight of the target protein, and then transferred to nitrocellulose sheets (NEN Life Science Products, Inc., Boston, MA, USA). After blocking, the blots were incubated with iNOS (1:1000; BD Bioscience, Franklin Lakes, NJ, USA, 610432), p-IKK α/β (1:1000; Genetex, Washington, DC, USA, GTX9039), p-IκB (1:1000; Genetex, GTX00967), nuclear NF-κB (1:500; Genetex, GTX102090), IL-1β (1:1000, Santacruz, Dallas, TX, USA, sc-127420), CD44 (1:500; proteintech, 15675-1-AP), nuclear CD44-ICD (1:500; Cosmobio, Tokyo, Japan, #KAL-K0604), GAPDH (1:5000; abcam, ab9485), β tubulin (1:1000; abcam, ab6046), and Lamin B1 (1:1000; abcam, ab16048) in 5% nonfat skim milk. After washing three times with Tris-buffered saline with 0.1% Tween^®®^ 20 detergent (TBST), the blots were incubated with anti-mice or anti-rabbit IgG conjugated with horseradish peroxidase (HRP) (abcam, Waltham, MA, USA) for 2 h. Immunoblots were developed using the enhanced chemiluminescence solution (Millipore, Burlington, MA, USA).

### 4.12. Immunohistochemistry

The liver tissue sections were deparaffinized, rehydrated, and then incubated with boiling citrate buffer (pH 6.0) for 20 min. After cooling to room temperature, the sections were incubated in hydrogen peroxide solution and blocking buffer for 20 min. After blocking, the sections were incubated with primary antibodies of target proteins (p-IKK α/β and p-IκB) overnight at 4 °C. The sections were washed with TBST 3 times, incubated with secondary antibodies, and then developed using a rabbit-specific HRP/DAB (ABC) detection IHC kit (abcam, USA). The sections were counterstained with 10% hematoxylin. After mounting using DPX, the slides were randomly chosen in five areas, and then the number of expression cells for each field was counted under a microscope (Nikon Eclipse T2, Tokyo, Japan).

### 4.13. Immunofluorescence

The liver tissue sections were deparaffinized and rehydrated through graded concentrations of ethanol and water. The sections were incubated with boiling citrate buffer (pH 6.0) for 20 min. After the sections cooled down to room temperature, they were incubated in blocking buffer (5% bovine serum albumin (BSA) in phosphate-buffered saline (PBS)) for 1 h and then incubated with a CD44 primary antibody overnight at 4 °C. Sections were washed with PBS 3 times and incubated with secondary antibodies and 4′,6-diamidino-2-phenylindole (DAPI) for 1 h at room temperature in the dark. Images were captured with a microscope (Nikon, Tokyo, Japan) under a fluorescence setup with appropriate filters.

### 4.14. Statistical Analysis

Data were expressed as mean ± standard deviation. Significant differences between the means of the two independent groups were analyzed using ANOVA (GraphPad Prism version 7, GraphPad Software, Inc., Boston, MA, USA). Statistical difference was set at *p* < 0.05.

## 5. Conclusions

This present study may be the first report to discover that CD44-ICD plays a crucial role in the sepsis-relative inflammatory response. We used DAPT, the specific inhibitor of CD44-ICD, to inhibit the expression of CD44-ICD and found that hepatic inflammation can be reduced. The role of CD44-ICD on hepatic inflammation during sepsis has never been investigated. Therefore, CD44-ICD may be a potential target to attenuate LPS-induced hepatic inflammation.

## Data Availability

The data will be available from the corresponding author(s).

## Supplementary Materials

The following supporting information can be downloaded at https://www.mdpi.com/article/10.3390/ijms25168907/s1.
