# Supplementary material for: Inhibiting CD44-ICD Attenuates LPS-Induced Initiation of Hepatic Inflammation in Septic Mice
Source: Int J Mol Sci. 2024 Aug 15;25(16):8907. doi: 10.3390/ijms25168907 (PMC11354311; doi:10.3390/ijms25168907)
Supplement: Supplementary file 1 [file ijms-25-08907-s001.zip › ijms-3139019-supplementary.pdf]

Supplementary Materials

Inhibiting CD44-ICD Attenuates LPS-Induced Initiation of Hepatic Inflammation in Septic Mice

Li-Hsuan Li, Dur-Zong Hsu, Victor Raj Mohan Chandrasekaran * and Ming-Yie Liu *

Department of Environmental and Occupational Health, College of Medicine, National Cheng Kung University, 138 Sheng-Li Road, Tainan 70428, Taiwan

* Correspondence: victorrajmohan@gs.ncku.edu.tw (V.R.M.C.); myliu@mail.ncku.edu.tw (M.-Y.L.); Tel.: +886-6-235-3535 (ext. 5808) (V.R.M.C. & M.-Y.L.); Fax: +886-6-275-2484 (V.R.M.C. & M.-Y.L.)

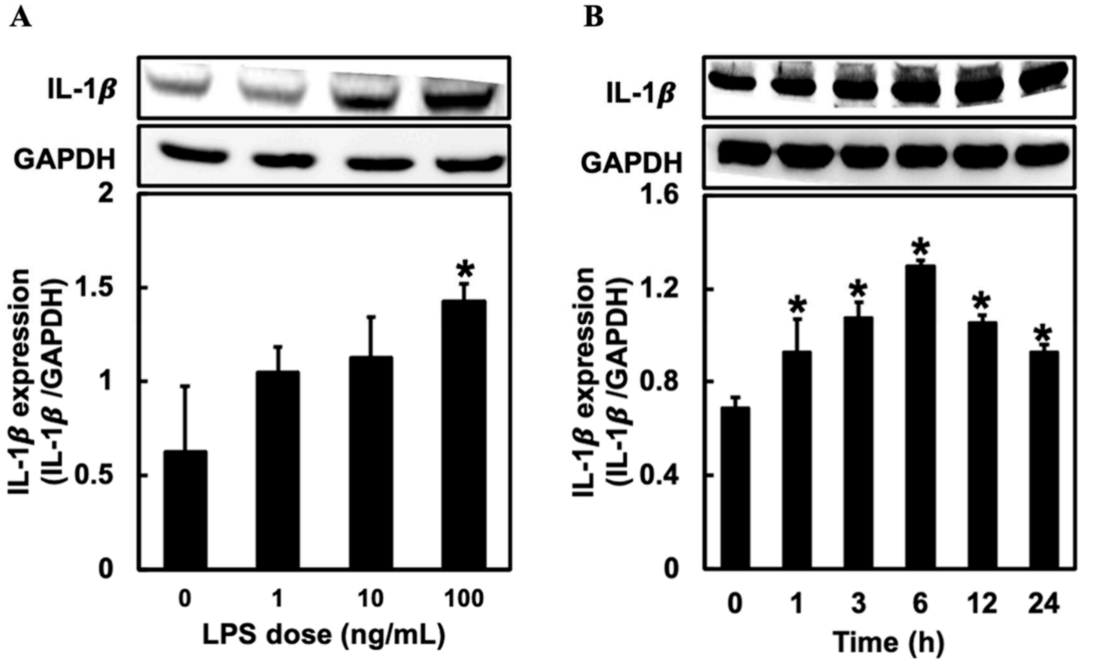

Supplementary Figure S1. The dose-response and time course effect of IL-1 β in LPS-induced inflammation in Chang cells. (**A**) Chang cells were divided into four groups of six cells in different doses of LPS, and (**B**) six groups of six in different times effects of LPS. Data are expressed as mean ± SD (n = 5). * $p < 0.05$ compared with 0 groups.
